# Hippocampal lipid differences in Alzheimer's disease: a human brain study using matrix‐assisted laser desorption/ionization‐imaging mass spectrometry

**DOI:** 10.1002/brb3.517

**Published:** 2016-07-14

**Authors:** Lakshini H. S. Mendis, Angus C. Grey, Richard L. M. Faull, Maurice A. Curtis

**Affiliations:** ^1^ Centre for Brain ResearchFaculty of Medical and Health ScienceUniversity of AucklandAucklandNew Zealand; ^2^ Department of Anatomy and Medical Imaging Faculty of Medical and Health ScienceUniversity of AucklandAucklandNew Zealand; ^3^ Department of PhysiologyFaculty of Medical and Health ScienceUniversity of AucklandAucklandNew Zealand

**Keywords:** Cornu Ammonis, dentate gyrus, human brain, neuroscience, receiver‐operator characteristic analysis

## Abstract

**Introduction:**

Alzheimer's disease (AD), the leading cause of dementia, is pathologically characterized by β‐amyloid plaques and tau tangles. However, there is also evidence of lipid dyshomeostasis‐mediated AD pathology. Given the structural diversity of lipids, mass spectrometry is a useful tool for studying lipid changes in AD. Although there have been a few studies investigating lipid changes in the human hippocampus in particular, there are few reports on how lipids change in each hippocampal subfield (e.g., Cornu Ammonis [CA] 1–4, dentate gyrus [DG] etc.). Since each subfield has its own function, we postulated that there could be lipid changes that are unique to each.

**Methods:**

We used matrix‐assisted laser desorption/ionization‐imaging mass spectrometry to investigate specific lipid changes in each subfield in AD. Data from the hippocampus region of six age‐ and gender‐matched normal and AD pairs were analyzed with SCiLS lab 2015b software (SCiLS GmbH, Germany; RRID:SCR_014426), using an analysis workflow developed in‐house. Hematoxylin, eosin, and luxol fast blue staining were used to precisely delineate each anatomical hippocampal subfield. Putative lipid identities, which were consistent with published data, were assigned using MS/MS.

**Results:**

Both positively and negatively charged lipid ion species were abundantly detected in normal and AD tissue. While the distribution pattern of lipids did not change in AD, the abundance of some lipids changed, consistent with trends that have been previously reported. However, our results indicated that the majority of these lipid changes specifically occur in the CA1 region. Additionally, there were many lipid changes that were specific to the DG.

**Conclusions:**

Matrix‐assisted laser desorption/ionization‐imaging mass spectrometry and our analysis workflow provide a novel method to investigate specific lipid changes in hippocampal subfields. Future work will focus on elucidating the role that specific lipid differences in each subfield play in AD pathogenesis.

## 1 Introduction

Although β‐amyloid (Aβ) plaques and tau tangles are the main pathological hallmarks of Alzheimer's disease (AD), Alois Alzheimer also noted the accumulation of fatty deposits in his paper first describing the disease (Alzheimer, 1907). The link between aberrant lipid metabolism and neurodegeneration in AD has been confirmed (Chan et al., 2012; Ellison, Beal, & Martin, 1987; Han, Holtzman, & McKeel, 2001; Han, Holtzman, McKeel, Kelley, & Morris, 2002; He, Huang, Li, Gong, & Schuchman, 2010; Jolles, Bothmer, Markerink, & Ravid, 1992; Jope, Song, Li, & Powers, 1994; Landman et al., 2006; Lange et al., 2008; Martın et al., 2010; Mulder et al., 2003; Nitsch et al., 1992; Pernber, Blennow, Bogdanovic, Månsson, & Blomqvist, 2012; Söderberg, Edlund, Kristensson, & Dallner, 1991; Svennerholm & Gottfries, 1994; Wells, Farooqui, Liss, & Horrocks, 1995).

Lipids found in the brain fall into three major categories: cholesterol, phospholipids, and sphingolipids (Jackson, Wang, & Woods, 2005). The phospholipids category includes the following classes of lipids: phosphatidylcholines (PCs), phosphatidylethanolamines (PEs), phosphatidylinositols (PIs), phosphatidylserines (PSs). The sphingolipids category contains sphingomyelin (SM), cerebrosides, ceramides (Cer), sulfatides (SFs), and gangliosides (GMs).

The fundamental role that phospholipids and sphingolipids play in membrane architecture is well defined. The polar headgroup and apolar fatty acid residue of these phospholipids allow marked amphilicity, leading to the characteristic formation of the lipid bilayer, which enables the maintenance of homeostasis (Schiller et al., 2004; Söderberg et al., 1991). Additionally, membrane microdomains rich in sphingolipids form lipid rafts that mediate compartmentalized cellular processes, as there is a clustering of receptors and signaling in these areas (Di Paolo & Kim, 2011). The role lipids play in intracellular signaling in the brain has also been well established. For instance, PIs are phosphorylated to produce phosphoinositides that can act as second messengers, in addition to mediating plasma membrane‐cytoskeleton interactions (Di Paolo & De Camilli, 2006). Thus, there has been a renewed interest in the neuromodulatory role that lipids play, particularly during cell proliferation, growth, and neuroprotection (Buccoliero & Futerman, 2003; Söderberg et al., 1991; Veloso, Astigarraga, et al., 2011; Veloso, Fernández, et al., 2011). Further, it has been reported that even limited alterations in phospholipid structure may have a considerable effect on function (Söderberg et al., 1991).

Many studies have detailed the dyshomeostasis of different lipid groups in AD. Specifically, PCs (Martın et al., 2010; Nitsch et al., 1992; Wells et al., 1995) and PEs (Ellison et al., 1987; Martın et al., 2010; Pettegrew, Panchalingam, Hamilton, & McClure, 2001; Wells et al., 1995) are decreased in AD. Both PCs and PEs also showed a variation in the ratio between saturated to unsaturated fatty acids in AD (Mulder et al., 2003; Söderberg et al., 1991). There is also a decrease in PIs, with disruptions to the production and signaling pathways of some phosphoinositides (Jope et al., 1994; Landman et al., 2006; Martın et al., 2010; Pettegrew et al., 2001; Prasad, Lovell, Yatin, Dhillon, & Markesbery, 1998; Stokes & Hawthorne, 1987; Wells et al., 1995). Additionally, Lange et al. (2008) reported a loss of PS asymmetry, with increased externalization of PSs to the outer leaflet of the lipid bilayer under oxidative stress. While early studies report an increase in SFs in AD (Majocha, Jungalwala, Rodenrys, & Marotta, 1989), there is now evidence of a significant SF depletion (up to 58% in white matter and up to 93% in gray matter), even at the earliest clinical stage of AD that was investigated (i.e., Clinical Dementia Rating 0.5; Han et al., 2002), with a consequent elevation in ceramide levels (Han, 2010; Han et al., 2002; He et al., 2010). GMs are also changed in AD, with an early study by Svennerholm and Gottfries (1994) reporting a decrease in GMs in the human hippocampus. However, more recently, there have been reports of elevated GM1 and GM2 in lipid rafts (Pernber et al., 2012), elevated GM3 (Chan et al., 2012) in the entorhinal cortex, and a decrease in b‐series GM alone, with a change in the composition of two particular GM1 species in the dentate gyrus (DG; Hirano‐Sakamaki et al., 2015). For an extensive review of the role lipid dyshomeostasis plays in mediating and modulating AD pathology see (Ariga, McDonald, & Robert, 2008; Di Paolo & Kim, 2011; Hartmann, Kuchenbecker, & Grimm, 2007; Yanagisawa, 2007). Finally, given that Cer, SM, and GMs are core constituents of lipid rafts, the change in the composition of these sphingolipids also impacts the function of these microdomains (Díaz et al., 2015; Fabelo et al., 2014). While pools of APP and presenilin are present in cell membranes, both in raft and nonraft regions, evidence that the amyloidogenic processing of amyloid precursor protein primarily occurs in lipid rafts is now well established and has been previously reviewed extensively (Cordy, Cordy, Hooper, & Turner, 2006; Di Paolo & Kim, 2011; Rushworth & Hooper, 2010). Thus, early changes in sphingolipids may play a role in the production and formation of Aβ plaques, driving AD pathogenesis. Aβ aggregation in lipid rafts also drives the accumulation of phosphorylated tau in these microdomains, at least in a mouse model of AD (Kawarabayashi et al., 2004).

Although there is now evidence that most, if not all, classes of lipids are implicated in the pathology of AD, few studies have focused specifically on the hippocampus, which is one of the main regions of atrophy in AD. Further, with the exception of Hirano‐Sakamaki et al. (2015), the majority of these findings have been based on traditional mass spectrometry or chromatography techniques, which can only provide crude anatomical detail (Kosicek & Hecimovic, 2013). Matrix‐assisted laser desorption/ionization (MALDI)‐imaging mass spectrometry (IMS), which was introduced in 1997 (Caprioli, Farmer, & Gile, 1997), offers an advantage over traditional mass spectrometry methods as it allows the visualization of the distribution of lipids, peptides, proteins, and drugs, in a tissue of interest (Cornett, Reyzer, Chaurand, & Caprioli, 2007). Using MALDI‐IMS, Veloso, Fernández, et al. (2011) were able to illustrate the anatomical variation in the distribution of lipids in the human hippocampus. However, there is little evidence of how these lipids change in the hippocampal subfields in AD. Thus, we optimized the MALDI tissue preparation to specifically detect lipids, using the 1,5‐diaminonaphthalene (DAN) matrix (Thomas, Charbonneau, Fournaise, & Chaurand, 2012), and conducted a spatially resolved analysis to find specific lipid changes in the hippocampal subfields on fresh tissue from normal and AD affected postmortem human brains.

## 2 Material and Methods

### 2.1 Chemicals and reagents

1,5‐Diaminonaphthalene, red phosphorous, and ammonium formate were purchased from Sigma‐Aldrich (St Louis, MO). Tissue‐Tek^®^ OCT compound 4583 was sourced from Sakura Finetek (Torrance, CA). Unless otherwise stated, all other reagents were purchased from Sigma‐Aldrich.

### 2.2 Tissue collection

Fresh, frozen postmortem human brain tissue, from the hippocampus block (hp2; in keeping with that described by Waldvogel, Curtis, Baer, Rees, & Faull, 2006) of each case, was obtained from the Neurological Foundation of New Zealand Human Brain Bank (University of Auckland, NZ). The tissue used for this study had been processed according to a detailed protocol, which has been previously published (Waldvogel et al., 2006, 2008), dissected into blocks, snap frozen on dry ice, and stored at −80°C. Table 1 provides in‐depth details about the six normal and six AD cases used in this study. Cases were age‐ and gender‐matched, and matched cases have been listed consecutively in each section. This coupling was important for our pair‐wise analysis. The use of this tissue was approved by the University of Auckland Human Participants Ethics Committee Ref No. 011654. All tissues were obtained with full informed consent of the families.

**Table 1:** Table summarizing age, gender, postmortem delay, cause of death, and pathology, for normal (*n* = 6) and Alzheimer's disease (AD; *n* = 6) cases used in this study

Case	Age (years)	Gender	Postmortem delay (hours)	Cause of death	Pathology
Normal					
H137	77	F	21	Coronary atherosclerosis	No significant histological abnormalities
H152	79	M	18	Congestive heart failure	No significant histological abnormalities
H169	81	M	24	Asphyxia	No significant histological abnormalities
H180	73	M	33	Ischemic heart disease	No significant histological abnormalities
H190	72	F	19	Ruptured myocardial infarction	No significant histological abnormalities
H238	63	F	16	Dissecting aortic aneurysm	No significant histological abnormalities
Alzheimer's disease					
AZ32	75	F	3	Bronchopneumonia	Braak: Unknown; CERAD: Probable Alzheimer'sA2 B1 C2
AZ80	77	M	4.5	Myocardial infarction	Braak: VI; CERAD: Definitive Alzheimer'sA3 B3 C3
AZ45	82	M	4.5	Pneumonia, stroke	Braak: Unknown; CERAD: Probable Alzheimer'sA1 B2 C2
AZ90	73	M	4	Gastrointestinal hemorrhage	Braak: IV; CERAD: Definitive Alzheimer'sA3 B3 C3
AZ72	70	F	7	Lung cancer	Braak: V; CERAD: Indicative of Alzheimer'sA0 B1 C3
AZ71	61	F	6	Severe dementia	Braak: VI; CERAD: Definitive Alzheimer'sA2 B3 C3

The age (mean ± *SD*) of the normal and AD cases were 74.2 ± 6.46 and 73 ± 7.13 years, respectively. The postmortem delay (mean ± *SD*) of the normal and AD cases were 21.83 ± 6.11 and 4.83 ± 1.43 h, respectively. The normal cases used in this study did not show significant histological abnormalities. The Braak and Braak stage and the Consortium to Establish a Registry for Alzheimer's disease (CERAD) score for the Alzheimer's disease cases used in this study are presented. The extent of atrophy (A; 0–3); neurofibrillary tangles (B; 0–3), and neuritic plaques (C; 0–3), for these cases are also given. Cases were age‐ and gender‐matched and matched cases have been listed consecutively in each section.

### 2.3 Tissue preparation

Twelve‐micrometer thick coronal sections, from the hp2 block, were cut at −20°C on a Leica Bright OTF5000 Cryostat (A‐M systems, USA), and thaw‐mounted on to pre‐cooled indium‐tin oxide‐coated MALDI glass slides (Hudson Surface Technology, USA). The slides were then dried for an hour in a dry‐seal, grease‐free desiccator (Jencons, USA), under vacuum, and then washed with ammonium formate as previously outlined by Angel, Spraggins, Baldwin, and Caprioli (2012) to reduce sodium and potassium adducted species, before matrix deposition. 1,5‐Diaminonaphthalene matrix was deposited, at 140°C for 5 min with a vacuum level of ~50 mTorr (Thomas et al., 2012), using the vacuum sublimation technique which has been published previously in detail (Hankin, Barkley, & Murphy, 2007).

### 2.4 MALDI imaging

Mass spectrometric analyses were performed at an accelerating voltage of +20 kV or −20 kV on a Bruker UltrafleXtreme MALDI‐TOF/TOF mass spectrometer (Bruker, Germany), equipped with a 2 kHz Smartbeam II™ UV MALDI laser and 60–70 μm laser beam size, operating in reflector mode. Delayed extraction parameters (120 ns) were optimized for signal intensity and mass resolution, and red phosphorous (1 mg/ml in ACN; Sigma‐Aldrich Chemistry, USA) was used as an external calibrant before data collection. The distance between raster points was set to 100 μm. Data were collected in the range of *m/z* 400–2,000. MALDI‐IMS data for each age‐ and gender‐matched normal and AD case were acquired in the same dataset. One section from each age‐ and gender‐matched normal and AD case was used to acquire data in negative ion mode, using 75 laser shots per spectrum, and another section from each case was used to acquire data in positive ion mode using 100 laser shots per spectrum. Since lipid distributions were found to be highly reproducible between sections from the same case (data not shown), one dataset from each matched pair was used for subsequent data analysis. Following data acquisition, DAN matrix was removed by 70% ethanol, and tissue sections were stained with hematoxylin and eosin (H&E) and luxol fast blue (LFB) for histological analysis.

### 2.5 Data analysis

**Figure 1:**
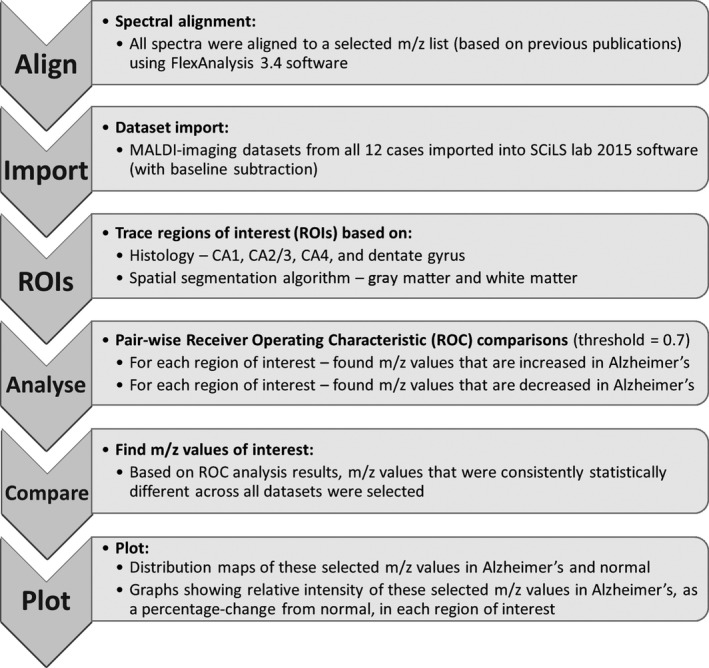
Data analysis workflow. Once data were acquired, spectra were aligned to a *m/z* list based on previous publications (Jackson et al., 2005, 2007; Veloso, Fernández, et al., 2011; Yuki et al., 2011), using FlexAnalysis 3.4 software. The datasets were then imported into SCiLS lab 2015b software (SCiLS GmbH, Germany; RRID:SCR_014426), and age‐ and gender‐matched normal and Alzheimer's cases were statistically analyzed using the receiver operating characteristic function. *m/z* values that were consistently statistically different across all six datasets were chosen. Distribution maps of the selected *m/z* values were generated using SCiLS lab 2015b software (SCiLS GmbH, Germany; RRID:SCR_014426). Graphs showing relative intensity changes in Alzheimer's disease, for each region of interest, were generated using GraphPad Prism version 6 for Windows (GraphPad Software, La Jolla, CA; RRID:SCR_00279)

Data were analyzed using the workflow outlined in Fig. 1. Raw spectra from all datasets were first aligned to a control *m/z* list based on previous publications on the mammalian brain lipidome (Jackson, Wang, & Woods, 2007; Jackson et al., 2005; Veloso, Fernández, et al., 2011; Yuki et al., 2011), using FlexAnalysis 3.4 software (Bruker Daltonik GmbH, Germany). Datasets were then imported into SCiLS lab 2015b software (SCiLS GmbH, Germany; RRID:SCR_014426), with a TopHat baseline removal, and normalized to total ion count. The spatial segmentation tool with edge‐preserving image denoising, which groups together areas that have a similar *m/z* profile, was used to differentiate white matter and gray matter for further analysis. A coregistered high‐resolution scan of the section stained with H&E and LFB was used to trace out regions of interest (ROI; CA1, CA2/3, CA4 and DG regions), based on their histological appearance. A receiver‐operator characteristic (ROC) analysis was done on each ROI of each age‐ and gender‐matched pair, to statistically analyze *m/z* values that were either increased or decreased in AD. The ROC analysis results were then evaluated to find *m/z* values that were consistently statistically increased or decreased, in each ROI, across all six datasets. Distribution maps of these selected *m/z* values were generated using SCiLS lab 2015b (SCiLS Gmbh; RRID:SCR_014426), with automatic hotspot removal and edge‐preserving weak image denoising. The relative intensity change in these selected *m/z* values in AD was calculated as a percentage (%) change from normal, and the graphs showing this change for each region were generated using GraphPad Prism version 6 for Windows (GraphPad Software, La Jolla, CA; RRID:SCR_00279).

### 2.6 Putative lipid identification

Lipid identifications were made in comparison with published mammalian lipid identifications where possible. For others, on‐tissue MALDI‐MS/MS, which was performed using a Bruker UltrafleXtreme MALDI‐TOF/TOF mass spectrometer (Bruker, Germany) and analyzed using the LIPID MAPS database (Fahy, Sud, Cotter, & Subramaniam, 2007; RRID:SCR_003817), was used. However, given the mass resolution of the MALDI‐TOF and the relatively wide precursor ion selection window for MALDI‐TOF/TOF analysis, product ion peaks of isobaric lipids were also present within some of the MS/MS spectra. Thus, liquid‐chromatography (LC)‐MS/MS, which was performed using a Thermo Finnigan LTQ‐FT (Linear Ion Trap‐Fourier Transform) mass spectrometer (Thermo Scientific, USA) and analyzed using LipidSearch software (Thermo Scientific), was used to validate MALDI‐MS/MS results where possible. The results of the MALDI‐MS/MS and LC‐MS/MS analysis are included as supplementary data. While structural assignments for some lipids could not be made due to a low abundance, those detected in the higher *m/z* range in positive ion mode could not be identified accurately using the LIPID MAPS database (RRID:SCR_003817) as the library is not yet complete and is being continuously updated (Han, 2016). Overall, putative lipid assignments have been made for 22 of the lipids that were differentially expressed in AD.

## 3 Results

### 3.1 Lipids in the human hippocampus

Depending on their class, different lipids can be protonated or deprotonated more easily. Therefore, we chose to acquire two datasets separately for each age‐ and gender‐matched pair, using negative and positive polarities, to maximize the range of lipids analyzed during this study. Deprotonated lipids are detected easily when the negative polarity is used during data acquisition. In contrast, using the positive polarity during data acquisition allows detection of protonated lipids. Sodium and potassium adducted species were reduced with an ammonium formate wash (Angel et al., 2012). We used the 1,5‐DAN MALDI matrix, since it has previously been shown to produce high‐quality lipid spectra in both instrument polarities (Thomas et al., 2012).

**Figure 2:**
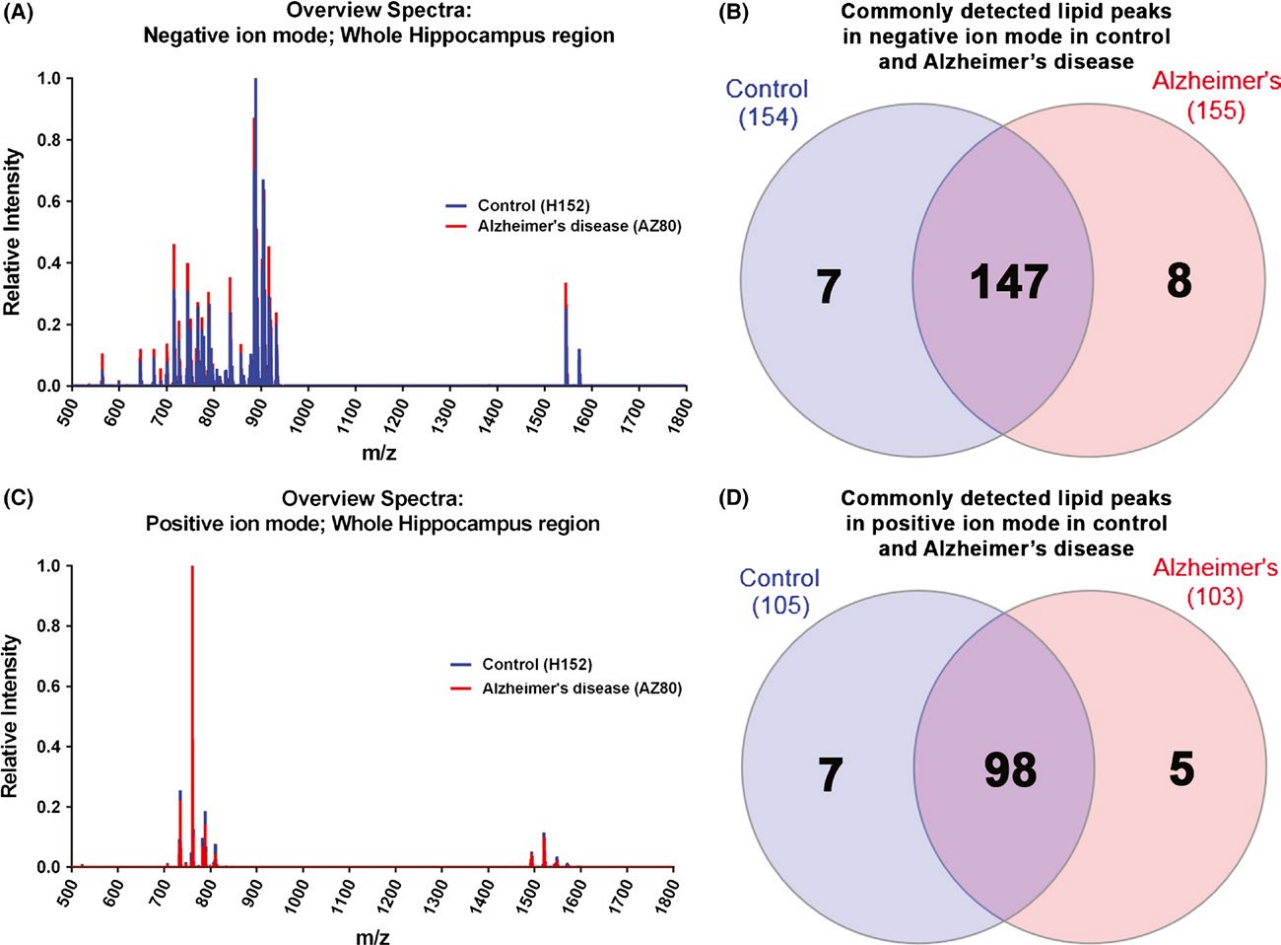
Overview of lipid spectra detected in the hippocampus. (A) Overall lipid spectrum acquired in negative ion mode, between *m/z* 500 to 1,800, from a normal (H152; blue) and Alzheimer's disease (AZ80; red) hippocampus. (B) A total of 154 and 155 lipid peaks were detected in normal and Alzheimer's disease cases, respectively. 147 lipid peaks were commonly found in both. (C) In contrast, fewer peaks are apparent in the overall lipid spectrum acquired in positive ion mode, from a normal (H152; blue) and Alzheimer's disease (AZ80; red) case. (D) 105 peaks were detected in normal and 103 peaks were detected in Alzheimer's disease, with 98 lipids commonly detected in both

Figure 2A shows the lipid profile of the postmortem human hippocampus, detected using negative ion mode, in normal (blue) and AD (red). Overall, 154 lipids peaks were detected in normal (blue) and 155 peaks were detected in AD, with 147 commonly detected in both (Fig. 2B). In contrast, fewer lipid peaks were detected using the positive ion mode, as seen in Fig. 2C, with 105 peaks detected in normal (blue) and 103 peaks detected in AD. Overall, 98 were commonly detected in both (Fig. 2D).

### 3.2 Differentially expressed negatively charged lipid species in AD

**Figure 3:**
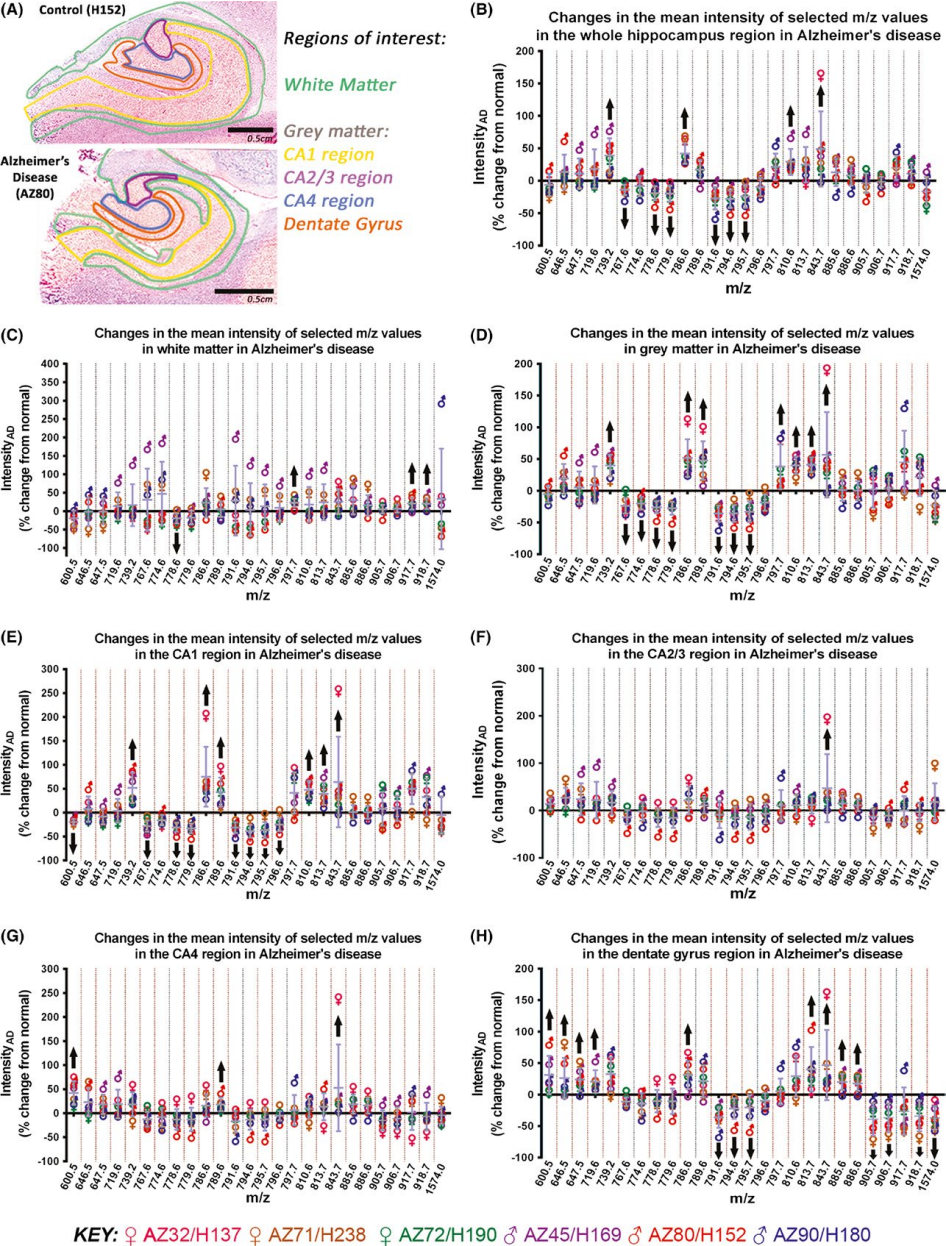
Mean intensity difference of selected *m/z* values, detected in negative ion mode, in Alzheimer's disease (as a percentage (%) change from normal). The regions of interest in the postmortem human hippocampus analyzed for this study, in a normal (H152) and an Alzheimer's disease case (AZ80) is shown in (A). (B–H) Graphs showing the mean intensity difference of selected *m/z* values in Alzheimer's disease, relative to intensity in normal human hippocampus, in the whole hippocampus (B), and white matter (C), gray matter (D), CA1 (E), CA2/3 (F), CA4 (G), and dentate gyrus (H) regions. *m/z* values were selected using pair‐wise receiver operating characteristic comparisons for each anatomical region. Black arrows indicate the lipid species that were consistently changed in all Alzheimer's disease cases

We used the analysis workflow developed in‐house (Fig. 1) to identify lipids that were differentially expressed in at least one of the subfields of the hippocampus (Fig. 3A) in AD. We detected 26 lipids that were differentially expressed either across the whole hippocampus region or in at least one of its subfields.

#### 3.2.1 Differentially expressed lipids across the whole hippocampus

There were ten lipids that were differentially expressed in AD across the whole hippocampus, of which the majority showed a relative decrease (Fig. 3B). These were *m/z* 767.6 (PE 38:4‐H^−^), 778.6 (PE 39:5‐H^−^/PPE 40:4‐H^−^), 779.6 (PG 37:6‐H^−^), 791.6 (PE 40:6‐H^−^), 794.6 (PE 40:4‐H^−^), and 795.7 (PG 38:5‐H^−^/PA 44:10‐H^−^). In contrast, we detected a relative increase in *m/z* 739.2, 786.6 (PS 36:2‐H^−^), 810.6 (PS 38:4‐H^−^), and 843.7.

#### 3.2.2 Differentially expressed lipids in white matter

While three lipid species *m/z* 797.7 (PG 38:4‐H^−^), 917.7, and 918.7 (SF 26:0 (0H)‐H^−^), were increased in white matter in AD, *m/z* 778.6 (PE 39:5‐H^−^/PPE 40:4‐H^−^) was the only lipid species that was decreased in this region (Fig. 3C).

#### 3.2.3 Differentially expressed lipids in gray matter

In contrast to the four lipids that were differentially expressed in white matter, 14 lipids were differentially expressed in gray matter, of which half were increased and half were decreased (Fig. 3D). The lipid species that were increased in gray matter include *m/z* 739.2, 786.6 (PS 36:2‐H^−^), 789.6 (SM 36:1‐H^−^), 797.7 (PG 38:4‐H^−^), 810.6 (PS 38:4‐H^−^), 813.7, and 843.7. In contrast, *m/z* 767.7 (PE 38:4‐H^−^), 774.6 (PE 39:7‐H^−^/PPE 40:6‐H^−^), 778.6 (PE 39:5‐H^−^/PPE 40:4‐H^−^), 779.6 (PG 37:6‐H^−^), 791.6 (PE 40:4‐H^−^), 794.6 (PE 40:4‐H^−^), and 795.7 (PG 38:5‐H^−^/PA 44:10‐H^−^) were decreased.

#### 3.2.4 Differentially expressed lipids in the Cornu Ammonis

Of the CA1, CA2/3, and CA4 regions, the CA1 region had the most changes in lipid expression in AD. There were eight lipids that were decreased in the CA1 region, of which six reflected the change seen in gray matter (Fig. 3E). The lipids that were decreased were *m/z* 600.5 (Cer 39:4‐H^−^), 767.6 (PE 38:4‐H^−^), 778.6 (PE 39:5‐H^−^/PPE 40:4‐H^−^), 779.6 (PG 37:6‐H^−^), 791.6 (PE 40:6‐H^−^), 794.6 (PE 40:4‐H^−^), 795.7 (PG 38:5‐H^−^/PA 44:10‐H^−^), 796.6 (PE 40:3‐H^−^). There were six lipids that were increased in the CA1 region, again reflecting the change seen in gray matter. These were *m/z* 739.2, 786.6 (PS 36:2‐H^−^), 789.6 (SM 36:1‐H^−^), 810.6 (PS 38:4‐H^−^), 813.7, and 843.7. There was only one lipid that was differentially expressed in the CA2/3 region in AD, and this was *m/z* 843.7, which was increased (Fig. 3F). The three lipids that were differentially expressed in the CA4 region in AD were also all increased (Fig. 3G). These were *m/z* 600.5 (Cer 29:4‐H^−^), 789.6 (SM 36:1‐H^−^), and 843.7.

#### 3.2.5 Differentially expressed lipids in the DG

There were 16 lipids that were differentially expressed in the DG in AD, of which eight were specifically only changed in this region (Fig. 3H). There were nine lipids that were increased. These were *m/z* 600.5 (Cer 39:4‐H^−^), 646.5 (Cer N24:1‐H^−^), 647.5 (PA 32:0‐H^−^), 719.6, 786.6 (PS 36:2‐H^−^), 813.7, 843.7, 885.6 (PI 38:4‐H^−^), and 886.7. The seven lipids that were decreased were *m/z* 791.6 (PE 40:6‐H^−^), 794.6 (PE 40:4‐H^−^), 795.7 (PG 38:5‐H^−^/PA 44:10‐H^−^), 905.7, 906.7 (SF 24:0 (0H)‐H^−^), 918.7 (SF 26:0 (0H)‐H^−^), and 1574.0 (GM1 d20:1/18:0).

The differential expression of these lipids in AD, in the subfields of the hippocampus, has been summarized in Table 2. We were able to assign putative lipid identities to 19 of the 26 *m/z* values that were detected by our analysis. Overall, when they were differentially expressed, generally PE and PG showed a decrease, while PS, PI, SF, and SM, were increased. Cer was increased in the DG, while the only GM was decreased in the same region.

**Table 2:** Summary of the relative change (i.e., an increase ↑ or decrease ↓; mean percentage change given in brackets) in the mean intensity of selected lipid species, which were detected in negative ion mode, in the Alzheimer's disease postmortem human hippocampus. An indication of how these lipids change in white matter and gray matter, as well as anatomically distinct areas (i.e., CA1, CA2/3, CA4 and dentate gyrus) is also included. Putative lipids assignments were based on MS/MS data (see Supporting information) and previous publications

Observed *m/z*	Lipid assignment (reference)	Hippocampus region						
		Whole region	White matter	Gray matter	CA1	CA2/3	CA4	Dentate gyrus
600.5	Cer 39:4‐H^−^ a				↓ (−19.5)		↑ (41.9)	↑ (31.1)
646.5	Cer N24:1‐H^−^ (Han et al., 2002)							↑ (26.1)
647.5	PA 32:0‐H^−^ b							↑ (23.9)
719.6								↑ (20.6)
739.2		↑ (38.7)		↑ (39.9)	↑ (51.7)			
767.6	PE 38:4‐H^−^ (Nunez et al., 2016)	↓ (−14.2)		↓ (−23.6)	↓ (−28.2)			
774.6	PE 39:7‐H^−^ cPPE 40:6‐H^−^ (Han et al., 2001)			↓ (−19.8)				
778.6	PE 39:5‐H^−^ cPPE 40:4‐H^−^ (Han et al., 2001)	↓ (−19.3)	↓ (−19.6)	↓ (−25.9)	↓ (−30.8)			
779.6	PG 37:6‐H^−^ c	↓ (−20.0)		↓ (−27.7)	↓ (−33.1)			
786.6	PS 36:2‐H^−^ c	↑ (41.9)		↑ (51.6)	↑ (75.0)			↑ (31.1)
789.6	SM 36:1‐H^−^ (Samhan‐Arias et al., 2012)			↑ (47.6)	↑ (42.7)		↑ (18.5)	
791.6	PE 40:6‐H^−^ (Han et al., 2001)	↓ (−27.0)		↓ (−36.0)	↓ (−31.4)			↓ (−36.4)
794.6	PE 40:4‐H^−^ c	↓ (−28.2)		↓ (−35.7)	↓ (−38.9)			↓ (−22.1)
795.7	PG 38:5‐H^−^/PA 44:10‐H^−^ b	↓ (−24.1)		↓ (−33.0)	↓ (−36.4)			↓ (−20.7)
796.6	PE 40:3‐H^−^ a				↓ (−24.9)			
797.7	PG 38:4‐H^−^ c		↑ (22.6)	↑ (38.0)				
810.6	PS 38:4‐H^−^ c (Dill et al., 2010)	↑ (29.1)		↑ (40.0)	↑ (47.8)			
813.7				↑ (41.0)	↑ (39.5)			↑ (40.3)
843.7		↑ (48.9)		↑ (56.7)	↑ (63.9)	↑ (11.8)	↑ (52.7)	↑ (46.8)
885.6	PI 38:4‐H^−^ (Dill et al., 2010; Jackson et al., 2005; Veloso, Astigarraga, et al., 2011; Veloso, Fernández, et al., 2011)							↑ (20.1)
886.7								↑ (17.8)
905.7								↓ (−37.2)
906.7	SF 24:0 (0H)‐H^−^ (Dill et al., 2010; Jackson et al., 2005; Veloso, Astigarraga, et al., 2011; Veloso, Fernández, et al., 2011; Yuki et al., 2011)							↓ (−36.8)
917.7			↑ (20.7)					
918.7	SF 26:0 (0H)‐H^−^ (Yuki et al., 2011)		↑ (20.3)					↓ (34.1)
1574.0	GM1 D20:1/18:0‐H^−^ (Ariga, Yu, Suzuki, Ando, & Miyatake, 1982; Chan et al., 2009; Whitehead et al., 2011)							↓ (−37.1)

Cer, ceramide; GM, ganglioside; PA, Phosphatidic acid; PC, Phosphatidylcholine; PE, Phosphatidylethanolamine; PG, Phosphatidylglycerol; PI, Phosphatidylinositol; PPE, Phosphatidylethanolamine plasmalogen; PS, Phosphatidylserine; SM, sphingomyelin.

a Putative lipid assignment based on LC‐MS/MS.

b Putative lipid assignment based on MALDI‐TOF‐TOF MS/MS data.

c Putative lipid assignment based on MALDI‐TOF‐TOF MS/MS and confirmed using LC‐MS/MS.

**Figure 4:**
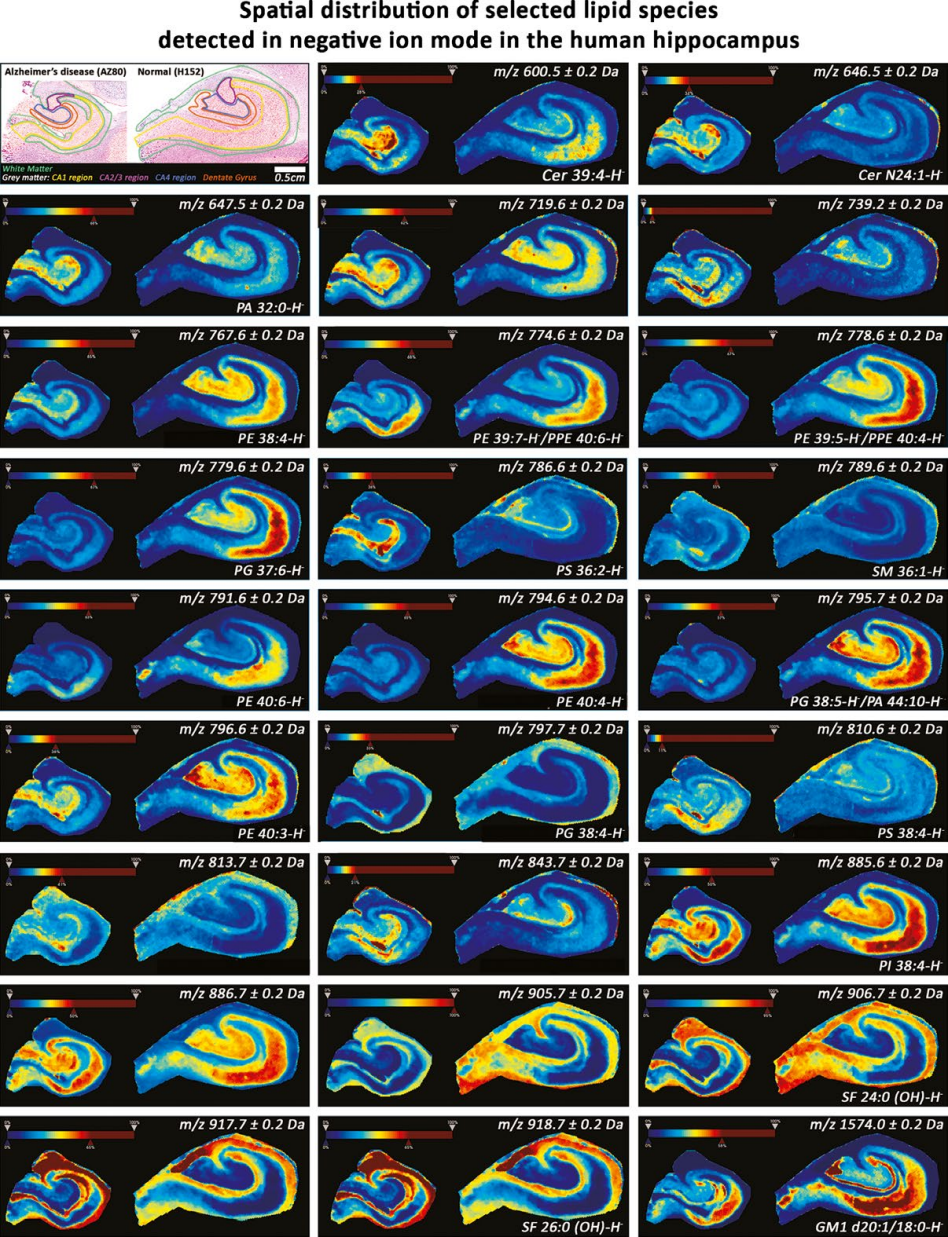
Spatial distribution of selected lipids detected in negative ion mode in the human hippocampus. Representative hippocampus sections (AZ80 and H152) stained with H&E (top‐left corner) showing white matter (green), and the gray matter regions, Cornu Ammonis 1 (CA1; yellow), CA2/3 (purple), CA4 (blue), and the dentate gyrus (orange). Edge‐preserving image denoising and automatic hotspot removal (see rainbow intensity color‐bar) was applied using SCiLS Lab 2015b software (SCiLS GmbH, Germany; RRID:SCR_014426). The spatial distance between adjacent spectra is 100 μm. Each image shows the distribution of a specific *m/z* value. Cer, ceramide; GM, ganglioside; PA, Phosphatidic acid; PC, Phosphatidylcholine; PE, Phosphatidylethanolamine; PG, Phosphatidylglycerol; PI, Phosphatidylinositol; PPE, Phosphatidylethanolamine plasmalogen; PS, Phosphatidylserine; SM, sphingomyelin

The distribution images clearly show the region‐specific differential expression of these lipids and their relative increase or decrease in AD (Fig. 4). The majority of these lipids, for example, *m/z* 600.5 (Cer 39:4‐H^−^), 794.6 (PE 40:4‐H^‐^), and 885.6 (PI 38:4‐H^−^) were expressed with a higher abundance in gray matter. However, even when expressed in gray matter, they did not show a uniform distribution. Many lipids, such as *m/z* 778.6 (PE 39:5‐H^−^/PPE 40:4‐H^−^), 794.6 6 (PE 40:4‐H^−^), and 885.6 (PI 38:4‐H^−^) were highly expressed in the CA1 region. Lipids that were abundantly expressed in the CA1 region were also usually expressed in the DG and CA4 region. However, others such as *m/z* 646.5 (Cer N24:1‐H^−^), 719.6, and 786.6 6 (PS 36:2‐H^−^), were highly expressed in the DG region alone, at least in AD. Six out of the 26 lipids detected in this analysis showed an inverse pattern of distribution, in that they were highly expressed in white matter. These were *m/z* 797.7 (PG 38:4‐H^−^), 813.7, 905.7, 906.7 (SF 24:0 (0H)‐H^−^), 917.7, and 918.7 (SF 26:0 (0H)‐H^−^).

### 3.3 Positively charged lipid species that were differentially expressed in the human hippocampus subfields in AD

**Figure 5:**
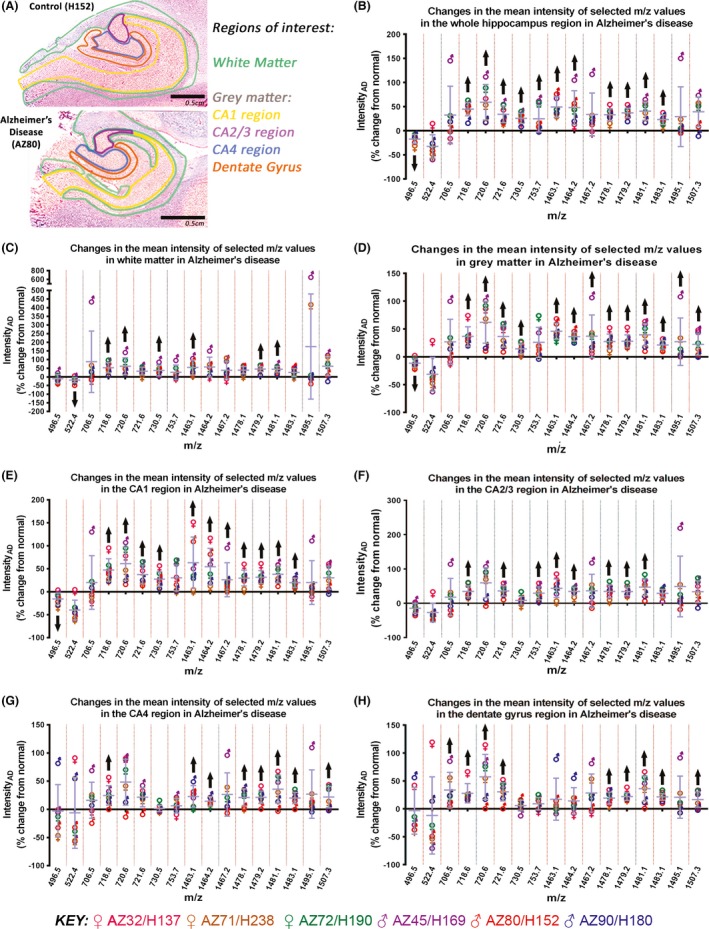
Mean intensity difference of selected *m/z* values, detected in positive ion mode, in Alzheimer's disease (as a % change from normal). The regions of interest in the postmortem human hippocampus analyzed in this study, in a normal (H152) and Alzheimer's disease case (AZ80) is shown in (A). (B–H) Graphs showing the mean intensity difference of selected *m/z* values in Alzheimer's disease, relative to intensity in normal human hippocampus, in the whole hippocampus (B), white matter (C), gray matter (D), CA1 (E), CA2/3 (F), CA4 (G), and dentate gyrus (H) regions. *m/z* values were selected using pair‐wise receiver operating characteristic comparisons for each anatomical region. Black arrows indicate the lipid species that were consistently changed in all Alzheimer's disease cases

Analysis of the positive ion mode datasets yielded 17 lipids that were differentially expressed, in at least one of the subfields of the hippocampus (Fig. 5A) in AD. However, where the same lipid was differentially expressed in AD, in more than one subfield, the lipid showed the same relative change (i.e., an increase or decrease) across all of those subfields.

#### 3.3.1 Differentially expressed lipids across the whole hippocampus

The only lipid that was decreased across the whole hippocampus was *m/z* 496.5 (Fig. 5B). In contrast, there was an increase in 11 lipids. These were *m/z* 718.6 (PE 34:1 + H^+^), 720.6 (PE 34:0 + H^+^), 721.6, 730.5, 753.7 (SM 36:1 + Na^+^), 1463.1, 1478.1, 1479.2, 1481.1, and 1483.1.

#### 3.3.2 Differentially expressed lipids in white matter

While *m/z* 522.4 was the only lipid that was decreased in white matter in AD, there were six lipids that were increased (Fig. 5C). These were *m/z* 718.6 (PE 34:1 + H^+^), 720.6 (PE 34:0 + H^+^), 730.5, 1463.1, 1479.2, and 1481.1.

#### 3.3.3 Differentially expressed lipids in gray matter

There were 14 lipids that were differentially expressed in AD in gray matter (Fig. 5D). The lipid detected at *m/z* 496.5 was the only one that showed a decrease. In contrast, *m/z* 718.6 (PE 34:1 + H^+^), 720.6 (PE 34:0 + H^+^), 721.6, 730.5, 1463.1, 1464.2, 1467.2, 1478.1, 1479.2, 1481.1, 1483.1, 1495.1, and 1507.3 were increased in gray matter in AD.

#### 3.3.4 Differentially expressed lipids in the Cornu Ammonis

Of the 12 lipids that were differentially expressed in the CA1 region in AD, *m/z* 496.5 was the only one that showed a decrease (Fig. 5E). The lipids that were increased were *m/z* 718.6 (PE 34:1 + H^+^), 720.6 (PE 34:0 + H^+^), 721.6, 730.5, 1463.1, 1464.2, 1467.2, 1478.1, 1479.2, 1481.1, and 1483.1.

All eight lipids that were differentially expressed in the CA2/3 region were increased in AD (Fig. 5F). These were *m/z* 718.6 (PE 34:1 + H^+^), 720.6 (PE 34:0 + H^+^), 721.6, 730.5, 1463.1, 1464.2, 1467.2, 1478.1, 1479.2, 1481.1, and 1483.1.

The CA4 region showed a similar pattern, with an increase in eight lipids in AD (Fig. 5G). The lipids that were increased in this region were *m/z* 718.6 (PE 34:1 + H^+^), 1463.1, 1464.2, 1478.1, 1479.2, 1481.1, 1483.1, and 1507.3.

#### 3.3.5 Differentially expressed lipids in the DG

**Figure 6:**
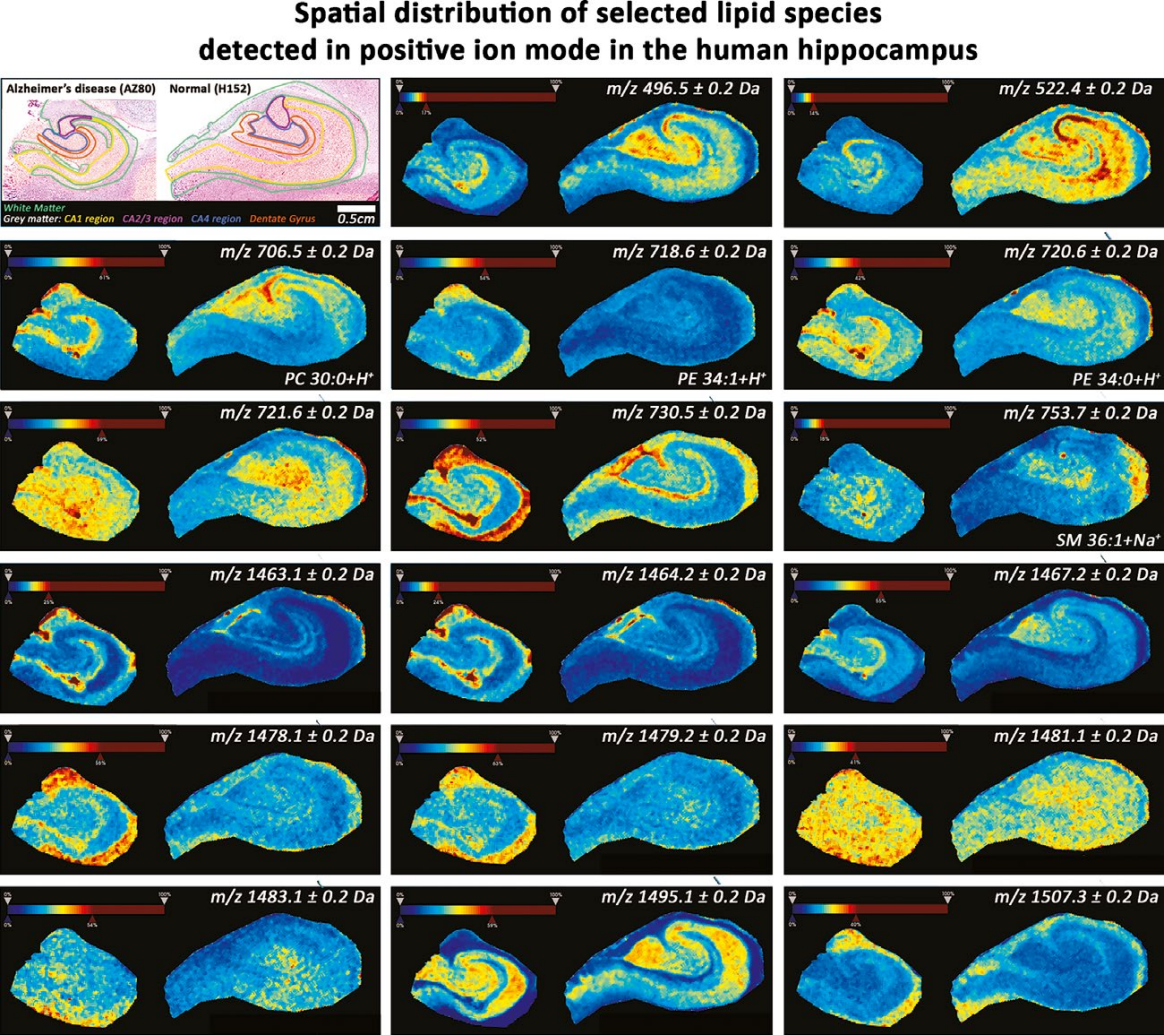
Spatial distribution of selected lipids detected in positive ion mode in the human hippocampus. Representative hippocampus sections (AZ80 and H152) stained with H&E (top‐left corner) showing white matter (green), and the gray matter regions, Cornu Ammonis 1 (CA1; yellow), CA2/3 (purple), CA4 (blue), and the dentate gyrus (orange). Edge‐preserving image denoising and automatic hotspot removal (see rainbow intensity color‐bar) has been applied. The spatial distance between adjacent spectra is 100 μm. Each image shows the distribution of specific *m/z* value. PC, Phosphatidylcholine; PE, Phosphatidylethanolamine; SM, sphingomyelin

There were nine lipids that were increased in the DG in AD (Fig. 5H). These were *m/z* 706.5 (PC 30:0 + H^+^), 718.6 (PE 34:1 + H^+^), 720.6 (PE 34:0 + H^+^), 721.6, 730.5, 1463.1, 1464.2, 1467.2, 1478.1, 1479.2, 1481.1, 1483.1, 1495.1, and 1507.3. Table 3 summarizes the differential expression of the lipids, detected in positive ion mode, in AD, across the different hippocampus subfields. The majority of lipids that were detected in positive ion mode were GMs, which were all increased in AD. The distribution of each lipid is seen in Fig. 6, with an indication of its differential expression within the same hippocampus, and the relative change in its abundance in AD. Generally, lipids were either expressed abundantly in gray matter, for example, *m/z* 496.5, 1467.2, and 1495.1, or in white matter, for example, *m/z* 1463.1, 1464.2, and 1507.3. Others, such as *m/z* 721.6, 1478.1, 1481.1, and 1483.1 showed a more homogenous expression through the hippocampus, with some hotspots in AD.

**Table 3:** Summary of the relative change (i.e., an increase ↑ or decrease ↓; mean percentage change given in brackets) in the mean intensity of selected lipid species, which were detected in positive ion mode, in the Alzheimer's disease postmortem human hippocampus. An indication of how these lipids change in white matter and gray matter, as well as anatomically distinct areas (i.e., CA1, CA2/3, CA4, and dentate gyrus) is also included. The mean percentage (%) change from normal is indicated in brackets. Putative lipids assignments were based on MS/MS data (see Supporting information) and previous publications

Observed *m/z*	Lipid assignment	Hippocampus region						
		Whole region	White matter	Gry matter	CA1	CA2/3	CA4	Dentate gyrus
496.5		↓ (−17.5)		↓ (−11.5)	↓ (−15.6)			
522.4			↓ (−18.9)					
706.5	PC 30:0 + H^+^ (Hicks, DeLong, Thomas, Samuel, & Cui, 2006)							↑ (33.6)
718.6	PE 34:1 + H^+^ a (Hicks et al., 2006)	↑ (44.5)	↑ (52.9)	↑ (36.6)	↑ (47.3)	↑ (34.6)	↑ (24.6)	↑ (28.0)
720.6	PE 34:0 + H^+^ (Hicks et al., 2006)	↑ (58.9)	↑ (61.2)	↑ (62.1)	↑ (61.2)			↑ (57.2)
721.6		↑ (34.0)		↑ (36.5)	↑ (37.5)	↑ (35.7)		↑ (30.5)
730.5		↑ (25.6)	↑ (37.5)	↑ (14.1)	↑ (27.6)			
753.7	SM 36:1 + Na^+^ (Fuchs, Nimptsch, & Schiller, 2008; Fujiwaki, Yamaguchi, Sukegawa, & Taketomi, 1999; Jackson et al., 2007)	↑ (24.7)				↑ (29.0)		
1463.1		↑ (49.0)	↑ (54.5)	↑ (45.9)	↑ (63.2)	↑ (43.6)	↑ (22.7)	
1464.2		↑ (47.8)		↑ (36.1)	↑ (54.5)	↑ (34.7)	↑ (14.3)	
1467.2				↑ (37.0)	↑ (26.2)			
1478.1		↑ (32.9)		↑ (27.3)	↑ (29.7)	↑ (34.4)	↑ (21.0)	↑ (19.2)
1479.2		↑ (36.7)	↑ (45.0)	↑ (27.7)	↑ (31.8)	↑ (34.1)	↑ (21.4)	↑ (21.8)
1481.1		↑ (40.5)	↑ (44.2)	↑ (39.5)	↑ (38.0)	↑ (47.0)	↑ (35.8)	↑ (36.2)
1483.1		↑ (22.9)		↑ (21.3)	↑ (19.7)		↑ (20.0)	↑ (21.2)
1495.1				↑ (27.1)				
1507.3				↑ (22.5)			↑ (21.8)	↑ (16.7)

PC, Phosphatidylcholine; PE, Phosphatidylethanolamine; SM, sphingomyelin.

a Putative lipid assignment based on LC‐MS/MS.

## 4 Discussion

This study demonstrates the anatomical compartments in which lipids were differentially expressed in the human hippocampus in AD. Due to the mass resolution limitations of the MALDI‐TOF, peaks could not always be assigned a single lipid identity (e.g., *m/z* 795.7 [PG 38:5‐H^−^/PA 44:10‐H^−^]), and may contain more than one lipid species. To confirm the identities of each lipid detected in our study, high mass resolution IMS equipment would be required. Nonetheless, this discussion focuses on the lipid assignments that we were able to putatively make using MS/MS and previously published results on the mammalian lipidome.

With the exception of PC 30:0 + H^+^, PE 34:1 + H^+^, PE 30:4 + H^+^, and PG 38:4‐H^−^, all lipid species belonging to the same class of lipid showed the same change in AD. For example, in AD, all the PS species detected in negative ion mode were increased. Additionally, if the same lipid species was changed in more than one hippocampus subfield, it was either increased or decreased across all subfields. Cer 39:4‐H^−^ and SF 26:0 (0H)‐H^−^ are the only lipid species that show a departure from this trend, as the former was decreased in the CA1 region, but increased in the CA4 and DG, while the latter was decreased in the DG but increased in white matter. It is however, important to note, that the extent to which the same lipid species was changed, which is indicated by the percentage change in Tables 2 and 3, differed in each subfield. This could largely be attributed to the variation in the abundance of these lipids in these subfields. Generally, there was an increase in ceramide, PC, PS, PI, SM, SFs, and GMs with a decrease in phosphatidylglycerols and PEs (except those detected in positive ion mode). With the exception of a few lipids, most of our results show trends that are consistent with previous work that have largely used traditional mass spectrometry and chromatography techniques to investigate lipid changes in AD (Chan et al., 2012; Cheng, Wang, Li, Cairns, & Han, 2013; Cutler et al., 2004; Ellison et al., 1987; Guan et al., 1999; Han et al., 2001, 2002; He et al., 2010; Jolles et al., 1992; Jope et al., 1994; Landman et al., 2006; Lange et al., 2008; Martın et al., 2010; Mulder et al., 2003; Nitsch et al., 1992; Pernber et al., 2012; Pettegrew et al., 2001; Söderberg et al., 1991; Svennerholm & Gottfries, 1994; Valdes‐Gonzalez et al., 2011; Wells et al., 1995). However, in contrast to previous work, we precisely delineated anatomical subfields in the hippocampus allowing us to determine specific lipid changes in AD in each region.

Of the 43 lipids that were changed in at least one hippocampus subfield in AD, only approximately half were detected when the hippocampus was analyzed as a whole. This highlights the merit in analyzing various subfields of a region of interest separately, using MALDI‐IMS. Additionally, with the exception of some lipids that were detected in positive ion mode that showed a homogenous distribution, most lipids were abundantly expressed either in gray matter or in white matter (Figs 4 and 6). There is also considerable heterogeneity in the expression of these lipids in the gray matter alone. This variation in lipid expression can be linked to the anatomical composition of each hippocampus subfield, particularly to gray and white matter. Since each hippocampus subfield has a distinct function, it is important to consider how lipid expression changes in each field, in order to link this change to AD pathogenesis.

Myelin is rich in SM and GM1 (O'Brien & Sampson, 1965; Pernber et al., 2012; Schnaar, Gerardy‐Schahn, & Hildebrandt, 2014; Svennerholm & Vanier, 1973). SM and GM1 are particularly concentrated in the outer leaflet of the membrane, where they are key constituents of lipid rafts (Farooqui, Horrocks, & Farooqui, 2000; Ramstedt & Slotte, 2002). Previously, in AD, an increase in SM had been reported (Bandaru et al., 2009; Pettegrew et al., 2001; Wells et al., 1995). Further, a significant decrease in ganglio‐series GMs (GT1b, GD1b, GD1a, GM1) in the frontal and temporal cortex, and an elevation in simple GMs like GM2, GM3, and GM4, have been reported (Kalanj, Kracun, Rosner, & Cosović, 1990; Kracun, Kalanj, Cosovic, & Talan‐Hranilovic, 1989). The increase in SM 36:1 and the decrease in GM1 seen in our study reflect these trends. Given the evidence that these changes occur very early during AD, accompanied by biophysical alterations and differential recruitment of amyloidogenic proteins to lipid rafts (Díaz et al., 2015; Fabelo et al., 2014), it is possible that these lipid changes may drive AD pathogenesis. Further, although we were unable to accurately identify lipids detected in the *m/z* 1,400–1,500 region in positive ion mode, given their proximity to expected *m/z* values, we suspect that some of these might be GMs. There is evidence that GM1 aggregation into clusters is accelerated in a cholesterol‐rich environment, as with aging and apolipoprotein E4 (apoE4) expression (Ariga et al., 2008; Pernber et al., 2012; Yanagisawa, 2007). Here, it can bind Aβ_1‐40_ and Aβ_1‐42_, which alters its conformation from random coils to more ordered structures, with increased β‐sheet content, leading to its aggregation (Kakio, Nishimoto, Yanagisawa, Kozutsumi, & Matsuzaki, 2002; Yanagisawa, 2007; Yanagisawa, Odaka, Suzuki, & Ihara, 1995). However, since we could only detect a decrease in GM1 d20:0/18:0 in the DG, further work specifically investigating other accurately identified GMs in the human hippocampus is needed to confirm the pathogenic role of GMs.

Sulfatides are also specifically expressed in myelin (Han et al., 2002; Yuki et al., 2011). The analysis of SFs in AD has produced conflicting results. Some groups report an increase in the average level of SFs in the AD brain (Majocha et al., 1989) and cerebrospinal fluid in vascular dementia (Fredman et al., 1992). Others have reported a significant depletion of up to 58% of SFs in white matter, even at the earliest clinical stage of AD they investigated (Clinical Dementia Rating 0.5;Han et al., 2002), with no change in the compositional distribution of hydoxylated and nonhydroxylated SFs between AD and normal (Yuki et al., 2011). However, there is no indication of how SF expression changes in the hippocampus, specifically. Our analysis indicated a 20% increase in SF 26:0 (0H)‐H^−^ in white matter in AD, with a depletion of SFs in other subfields, namely the DG. There is evidence that alterations in apoE‐mediated SF trafficking lead to this change in SF expression in AD (Han, 2010). The change in SF expression will affect its normal function, which includes myelin formation and maintenance, oligodendrocyte differentiation, myelin‐associated axon outgrowth, and glial‐axon signaling (Han et al., 2002; Takahashi & Suzuki, 2012).

The most abundant types of glycerophospholipids found in neural membranes are PCs, PEs, PSs, and PIs. Phosphatidylglycerols and phosphatidic acids, the main precursor of all neural membrane glycerophospholipids, are also found here, albeit with lower abundance (Farooqui et al., 2000). When gray matter was analyzed separately, many of the species detected that were changed in AD belonged to these classes of lipids. The lipids that were changed in gray matter were often specifically changed in just the CA1 region too. Additionally, of all Cornu Ammonis (CA) fields, the CA1 region yielded the majority of lipid changes. The CA1 region is considered the main output of the hippocampus, by the way of the alveus and then the fimbria (Duvernoy, Cattin, & Risold, 2013). Thus, it is a vital link in the intrahippocampal circuitry, which is crucial for memory formation (Duvernoy et al., 2013). In AD, with the exception of PE 34:1 + H^+^, which was increased in the CA1 region, there was a decrease in PEs, including PE 40:6‐H^−^, whose identification has been confirmed in the human brain before (Han et al., 2001). This PE decrease reflect previous findings (Chan et al., 2012; Ellison et al., 1987; Han et al., 2001; Kosicek & Hecimovic, 2013; Martın et al., 2010; Pettegrew et al., 2001; Wells et al., 1995). PEs are often linked to polyunsaturated fatty acids (PUFA), especially docosahexaenoic acid, which are highly oxidizable (Butterfield & Lauderback, 2002; González‐Domínguez, García‐Barrera, & Gómez‐Ariza, 2014; Hartmann et al., 2007). Thus, in AD, where there is increased oxidative stress, PUFAs serve as substrate for lipid peroxidation (Butterfield & Lauderback, 2002). In addition to leading to membrane destabilization, this process also generates aldehydes such as 4‐hydroxynonenal, which in turn can oxidize other proteins and inhibit glycolysis, driving AD pathogenesis (González‐Domínguez et al., 2014).

In contrast to the decrease in PEs, other lipids were increased in the CA1 region in AD. PS 36:2‐H^−^ showed the greatest change with a 75% increase. The PS content of gray matter increases from birth to the age of 80 (Glade & Smith, 2015; Vance & Tasseva, 2013), and under normal physiological conditions, it is located in the inner‐cytosolic leaf of membrane. However, during the early stages of apoptosis, PS is translocated to the outer layer of the membrane, where it can serve as an active signal for phagocytosis (Glade & Smith, 2015; Vance & Tasseva, 2013). Given the increased apoptosis in the CA1 region in AD, this may explain the elevated abundance of PS 36:2‐H^−^ in this region.

Finally, in contrast to the other subfields, there were a number of lipid species that were changed in DG alone. Of these lipids, the identification of PI 38:4‐H^−^, SF 24:0 (0H)‐H^−^, and Cer N24:1‐H^−^ have been reported previously (Dill et al., 2010; Han et al., 2002; Veloso, Fernández, et al., 2011; Yuki et al., 2011).

The 20% increase in PI 38:4‐H^−^ in the DG does not reflect previously reported decreases in PI in the AD human brain (Stokes & Hawthorne, 1987). Nonetheless, it may be a change which is specific to the DG that remains to be confirmed. We speculate that it may be a reflection of the reduction in PI kinases (Jolles et al., 1992) and the impaired phosphoinositides hydrolysis in AD (Jope et al., 1994). The role phosphoinositides play in regulation and membrane dynamics, which will be affected in AD, have been previously extensively reviewed by Di Paolo and De Camilli (2006) and Frere, Chang‐Ileto, and Di Paolo (2012).

While we did not analyze if there was a compositional difference between hydroxylated and nonhydroxylated SF species like Yuki et al. (2011), our analysis indicated several hydroxylated SFs that were decreased in AD in the DG alone, consistent with previous findings (Han et al., 2002; He et al., 2010). Since hydroxylated SFs are highly expressed in oligodendrocytes in gray matter, as mentioned previously, this decrease may affect oligodendrocyte differentiation, myelin formation and maintenance, myelin‐associated axon outgrowth, and glial‐axon signaling (Han et al., 2002; Takahashi & Suzuki, 2012). The decrease in SFs also consequently elevates ceramide levels in AD (Han et al., 2002; He et al., 2010; Hejazi et al., 2011; Puglielli, Ellis, Saunders, & Kovacs, 2003), which our results also reflected. Ceramide is thought to drive AD pathogenesis by playing a role in the execution of apoptosis (Hannun & Obeid, 2008; Hejazi et al., 2011), and stabilizing the β‐site amyloid precursor protein cleaving enzyme 1 (*BACE1*), consequently promoting Aβ biogenesis (Puglielli et al., 2003).

However, since work focusing on lipid changes in the postmortem human DG in AD is scarce, further work needs to be done to elucidate if these lipids play a specific role in driving DG function. Additionally, the effect their changed expression in AD has on the DG should also be evaluated. Finally, it remains to be determined if the observed lipid changes are a consequence or driver of AD pathogenesis.

Overall, we believe that the use of MALDI‐IMS and our subsequent analysis workflow provides a novel method to investigate changes in lipid expression in subfields of a region of interest. The advantage of using this approach is the ability to study precisely delineated subfields, based on their anatomical or chemical similarity. Now that we have identified lipids that show a relative change in the human AD hippocampus, we can target these lipids to quantify this change specifically in white matter, the CA1 and DG, which showed the greatest changes, to increase our understanding of their role in AD pathology. Finally, we believe that our analysis workflow can be applied to investigate metabolite and protein changes in precisely delineated anatomical subfields too.

## Funding Information

Health Research Council (3627373) and University of Auckland (lmen018 PReSS Account).

## Conflict of Interest

None declared.
