# Supplementary material for: Hippocampal lipid differences in Alzheimer's disease: a human brain study using matrix‐assisted laser desorption/ionization‐imaging mass spectrometry
Source: Brain Behav. 2016 Jul 14;6(10):e00517. doi: 10.1002/brb3.517 (PMC5064331; doi:10.1002/brb3.517)
Supplement: Supplementary file 1 [file BRB3-6-e00517-s001.pdf]

Supplementary information for:

Hippocampal lipid differences in Alzheimer's disease: A human brain study using Matrix-Assisted Laser Desorption/Ionization-Imaging Mass Spectrometry

Lakshini H. S. Mendis[1,2], Angus C. Grey[1,3], Richard L. M. Faull[1,2], Maurice A. Curtis[1,2]

1. Centre for Brain Research, Faculty of Medical and Health Science, University of Auckland, Private Bag 92019, Auckland, New Zealand
2. Department of Anatomy and Medical Imaging, Faculty of Medical and Health Science, University of Auckland, Private Bag 92019, Auckland, New Zealand
3. Department of Physiology, Faculty of Medical and Health Science, University of Auckland, Private Bag 92019, Auckland, New Zealand

Address reprint requests to:

[1] Associate Professor Maurice Curtis

E-mail address: m.curtis@auckland.ac.nz; Tel.: +64 9 3737599 ext 86999; Fax: +64 9 3737484;

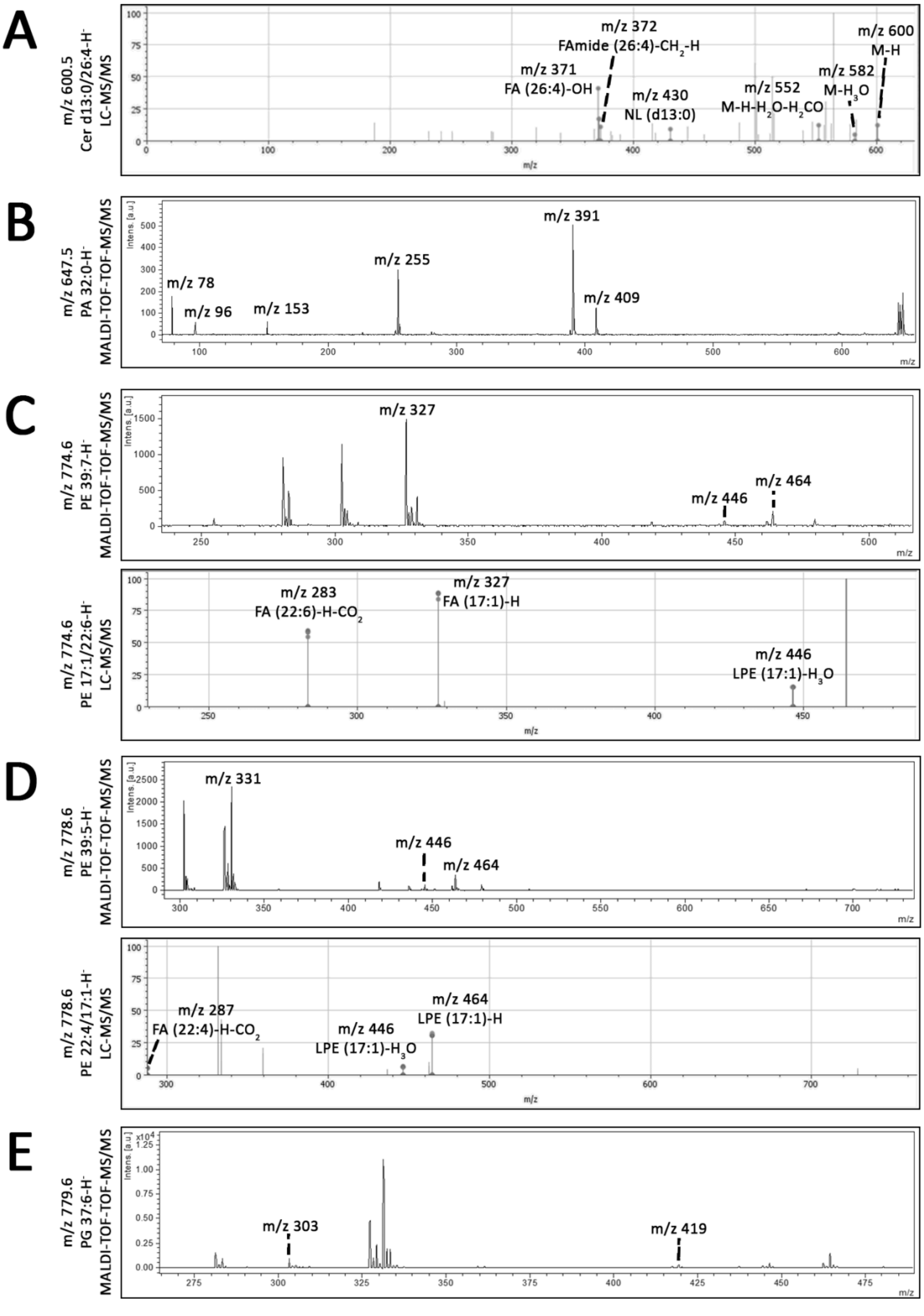

Figure S1: MS/MS spectra of selected m/z lipid species. Figure showing MS/MS spectra, acquired using MALDI-TOF-TOF and/or LC-MS/MS (as indicated), and analyzed using the LIPID MAPS database (Fahy et al., 2007) or LipidSearch software (Thermo Scientific, USA), respectively. (A) m/z 600.5, (B) m/z 647.5, (C) m/z 774.6, (D) m/z 228.6, and (E) m/z 779.6. The matched output from the LIPID MAPS database for the MALDI-TOF-TOF MS/MS data is shown in Table S1.

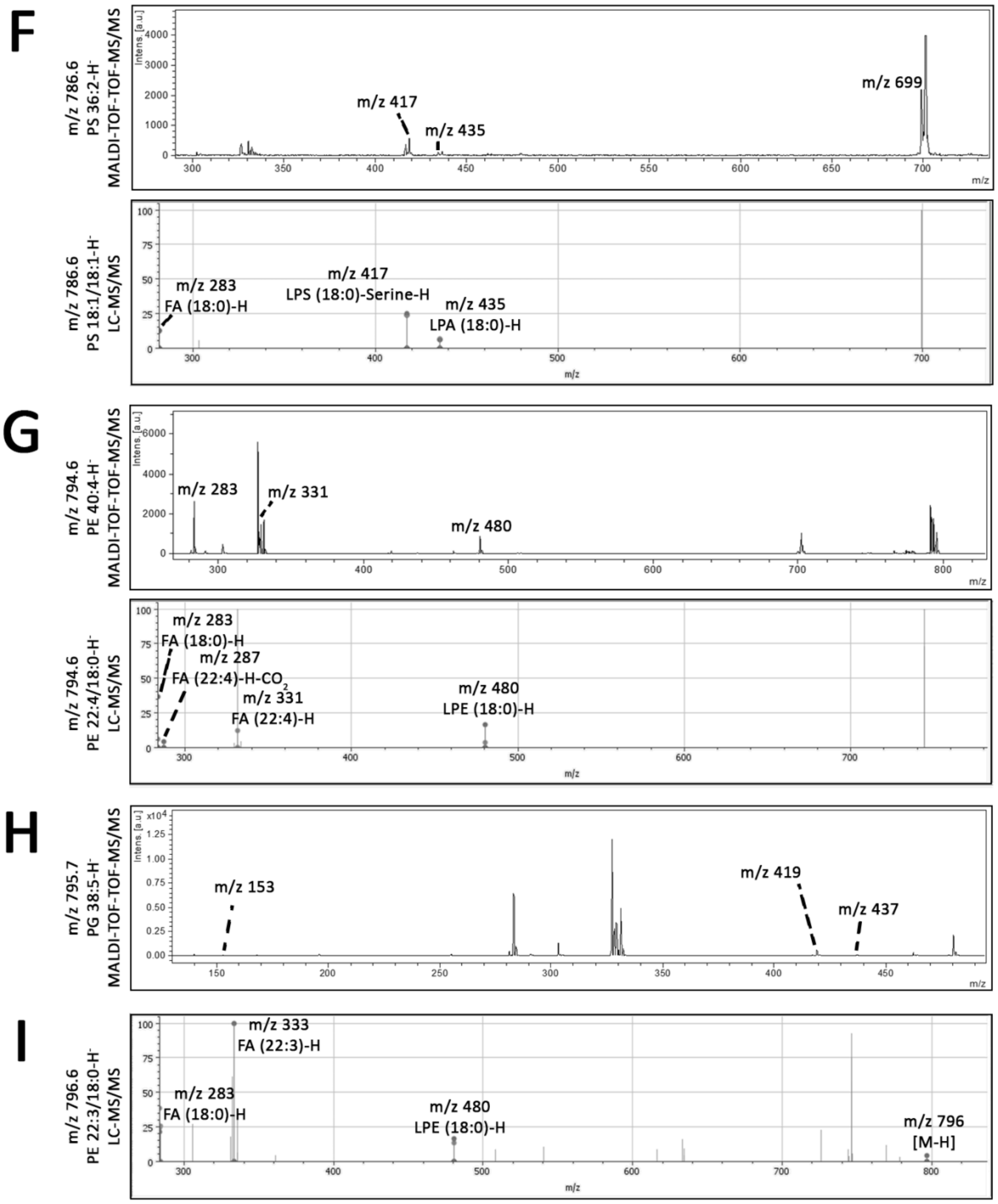

Figure S1 (continued): MS/MS spectra of selected m/z lipid species. MS/MS spectra, acquired using MALDI-TOF-TOF and/or LC-MS/MS (as indicated), and analyzed using the LIPID MAPS database (Fahy et al., 2007) or LipidSearch software (Thermo Scientific, USA), respectively. (F) m/z 786.6, (G) m/z 794.6, (H) m/z 795.7, and (I) 796.6. The matched output from the LIPID MAPS database for the MALDI-TOF-TOF MS/MS data is shown in Table S1.

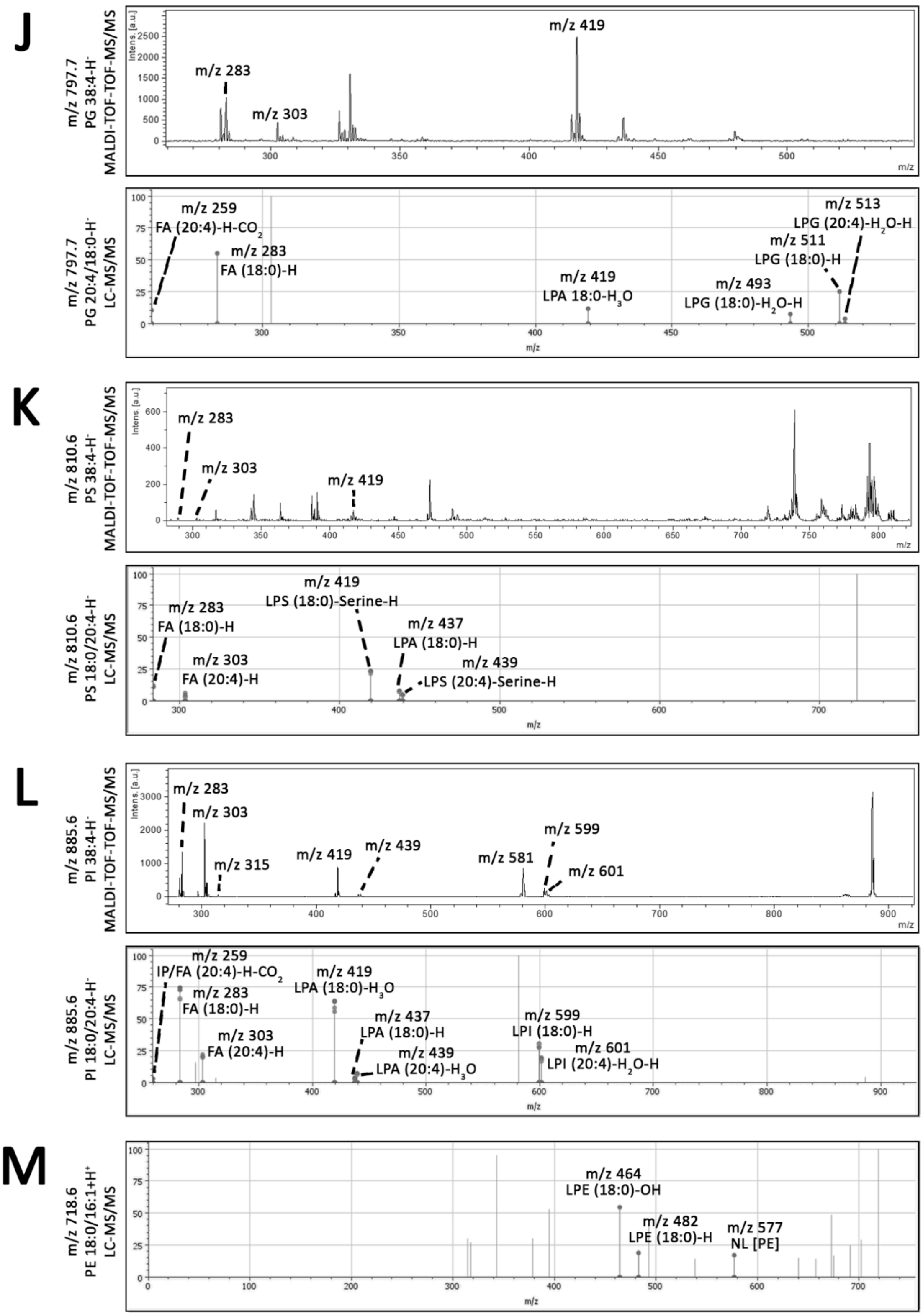

Figure S1 (continued): MS/MS spectra of selected m/z lipid species. MS/MS spectra, acquired using MALDI-TOF-TOF and/or LC-MS/MS (as indicated), and analyzed using the LIPID MAPS database (Fahy et al., 2007) or LipidSearch software (Thermo Scientific, USA), respectively. (J) m/z 797.7, (K) m/z 810.6, (L) m/z 885.6, and (M) m/z 718.6 (detected in positive ion mode). The matched output from the LIPID MAPS database for the MALDI-TOF-TOF MS/MS data is shown in Table S1.

Table S1: A summary of the matched output from LIPID MAPS (Fahy et al., 2007) for the product ions generated using MALDI-TOF/TOF-MS/MS, for the m/z species shown in Figure S1.

Lipid Species	MS/MS Product ions	LIPID MAPS database output
**m/z 647.5**	m/z 78	PO_3-ion
**PA 32:0-H$^-$**	m/z 96	H_2PO_4-ion
(Figure S1B)	m/z 153	Glycerol-3-phosphate ion with loss of H_2O
	m/z 255	sn1 RCOO-ion
		sn2 RCOO- ion
	m/z 391	Neutral loss of sn1 RCOOH group from [M-H]$^-$
		Neutral loss of sn2 RCOOH group from [M-H]$^-$
	m/z 409	Loss of sn1 acyl chain as RCOOH group from [M-H]$^-$
		Loss of sn2 acyl chain as RCOOH group from [M-H]$^-$
**m/z 774.6**	m/z 283	Loss of CO_2 from sn2 RCOO$^-$ ion (PUFA)
**PE 39:7-H$^-$**	m/z 327	sn2 RCOO- ion
(Figure S1C)	m/z 446	Neutral loss of sn2 RCOOH group from [M-H]$^-$
	m/z 464	Loss of sn2 acyl chain as ketene (RCH=C=O) from [M-H]$^-$
**m/z 778.6**	m/z 331	sn1 RCOO- ion
**PE 39:5-H$^-$**	m/z 446	Neutral loss of sn1 RCOOH group from [M-H}$^-$
(Figure S1D)	m/z 464	Loss of sn1 acyl chain as ketene (RCH=C=O) from [M-H]$^-$
**m/z 779.6**		
**PG 37:6-H$^-$**		
17:2/20:4	m/z 303	sn1/2 RCOO- ion
OR	m/z 419	Loss of sn1/2 acyl chain as ketene (RCH=C=O) and glycerol from [M-H]$^-$
22:6/15:0	m/z 327	sn1 RCOO$^-$ ion
(Figure S1E)	m/z 283	Loss of CO_2 from sn1 RCOO$^-$ ion
**m/z 786.6**	m/z 417	Neutral loss of sn1/2 RCOOH group and serine from [M-H]$^-$
**PS 36:2-H$^-$**	m/z 435	Loss off sn1/2 acyl chain as ketene (RCH=C=O) and serine from [M-H]$^-$
(Figure S1F)	m/z 699	Loss of serine from precursor ion
**m/z 794.6**	m/z 283	sn2 RCOO- ion
**PE 40:4-H$^-$**	m/z 331	sn1 RCOO- ion
(Figure S1G)	m/z 480	loss of sn1 acyl chain as ketene (RCH=C=O) from [M-H]$^-$

Table S1 (continued): A summary of the matched output from LIPID MAPS (Fahy et al., 2007) for the product ions generated using MALDI-TOF/TOF-MS/MS, for the m/z species shown in Figure S1.

Lipid Species	MS/MS Product ions	LIPID MAPS database output
**m/z 795.7**	m/z 153	Glycerol-3-phosphate ion with loss of H_2O
**PG 38:5-H$^-$**	m/z 283	sn1 RCOO$^-$ ion
	m/z 419	Neutral loss of sn1/2 RCOOH group and glycerol from [M-H]$^-$
	m/z 437	Neutral loss of sn1/2 RCOOH group and glycerol from [M-H]$^-$
**PA 44:10-H$^-$**		Loss of sn1/2 acyl chain and glycerol from [M-H]$^-$
(Figure S1H)	m/z 153	Glycerol-3-phosphate ion with loss of H_2O
	m/z 283	Loss of CO_2 from sn1 RCOO$^-$ ion (PUFA)
	m/z 331	sn2 RCOO$^-$ ion
	m/z 327	sn1 RCOO$^-$ ion
**m/z 797.7**	m/z 283	sn2 RCOO- ion
**PG 38:4-H$^-$**	m/z 303	sn1 RCOO- ion
(Figure S1J)	m/z 419	Neutral loss of sn1 RCOOH group and glycerol from [M-H]$^-$
**m/z 810.6**	m/z 283	sn1 RCOO- ion
**PS 38:4-H$^-$**	m/z 303	sn2 RCOO- ion
(Figure S1K)	m/z 419	Neutral loss of sn1 RCOOH group and glycerol from [M-H]$^-$
**m/z 885.6**	m/z 283	sn1 RCOO- ion
**PI 38:4-H$^-$**	m/z 303	sn2 RCOO- ion
(Figure S1L)	m/z 315	Glycero-phosphoinositol-$2H_2O$
	m/z 419	Neutral loss of sn2 RCOOH group and inositol from [M-H]$^-$
	m/z 439	Neutral loss of sn1 RCOOH group and inositol from [M-H]$^-$
	m/z 581	Neutral loss of sn2 RCOOH group from [M-H]$^-$
	m/z 599	Loss of sn2 acyl chain as ketene (RCH=C=O) from [M-H]$^-$
	m/z 601	Neutral loss of sn1 RCOOH group from [M-H]$^-$

Reference:

Fahy E, Sud M, Cotter D, Subramaniam S (2007) LIPID MAPS online tools for lipid research. Nucleic Acids Research 35:W606-W612.
